# The criterion of development of processes of the self-organization of subsystems of the second level in tribosystems of diesel engine

**DOI:** 10.1038/s41598-023-33015-3

**Published:** 2023-04-07

**Authors:** Denys Baranovskyi, Sergey Myamlin

**Affiliations:** 1 Faculty of Mechanics and Technology, Rzeszow University of Technology, Ul. Kwiatkowskiego 4, 37-450 Stalowa Wola, Poland; 2 Department of Development and Technical Policy, JSC “Ukrainian Railway”, Jerzy Giedroyc Str. 5, Kyiv, 03150 Ukraine

**Keywords:** Engineering, Materials science

## Abstract

The paper shows the possibilities of processes in the tribosystems of diesel engines, ensuring the development of self-organization processes in them. The criterion for the possibility of development of processes of the self-organization of subsystems of the second level shows that in a real irreversible process there is a decrease in the flow of mechanical energy. The paper considers three cases of the possibility of developing the processes of self-organization of subsystems of the second level on the example of the tribosystem “crankshaft-insert” for the operating conditions of a diesel engine 10D100. It was determined that in order to reduce the wear rate of the tribosystems of diesel engine it is necessary to provide the flow of the energy-mass transfer process on their contacting surfaces of friction by the gradients of chemical potentials and dislocation density of the interacting materials. The obtained expression is the criterion of possibility of development of processes of the self-organization of subsystems of the second level which indicates that the system would lose the stability, if the density of mobile dislocations or the wear rate of the tribosystems of diesel engine increases.

## Introduction

Self-organization is a fundamental phenomenon of nature and the essence of it is that under the influence of external disturbances any thermodynamically open nonlinear system is rebuilt so that its reaction maximally compensates for the reason that caused such internal change^1–3^. In living nature the processes of self-organization are the adaptation and evolution of biological objects corresponding to the changes in external conditions^3–7^. In engineering, the processes of self-organization are most clearly manifested in the tribosystems (TS) with the formation of secondary structures in friction and wear^7–12^.

The processes of friction and wear are realized through the presence of gradients of temperature, voltage, concentration of chemical elements and dislocation density^3,10,13^. Friction and wear act as unbalanced thermodynamic processes, so the occurrence of self-organization processes in them is inevitable. The following processes may be included in the internal manifestation of self-organization in the TS^1,3,14–16^:

- The formation of secondary structures to prevent the process of wear;
- The formation of balanced roughness to reduce wear;
- The increase the real contact area to reduce the wear rate;
- The realization of effect of selective transfer to insure the unworn state.

The external manifestation of self-organization is the reduction and stabilization of almost all the energy, strength and tribological parameters of the process of friction and wear^3,9,10,17–19^. As a consequence, the reduce of the time and the coefficient of friction, the temperature in the zone of friction, wear rate, etc. The general consistent pattern of manifestation of self-organization can be analyzed from the positions of system approach.

In many works on friction and wear of TS^9,20–24^, the results of studies of the course of self-organization processes in subsystems of the first, second and third levels are presented. However, the authors did not find studies of self-organization processes in subsystems of the second level in the TS of diesel engines. Therefore, the aim of the work is to establish the criterion of development of processes of the self-organization of subsystems of the second level in TS of diesel engine.

## Materials and research methods

In the process of studying the possibility of developing processes of self-organization of subsystems of the second level in TS of diesel engine, theories of thermodynamics of non-stationary processes, solid state physics, wear theory and the fundamentals of mechanics were used. To solve the differential equation of the energy-mass transfer, a numerical calculation technique was used.

The object of research is the processes of the self-organization of subsystems of the second level in TS of diesel engine.

The subject of research is the criterion of development of processes of the self-organization of subsystems of the second level in TS of diesel engine.

Research hypothesis is based on the Glansdorff–Prigozhin criterion and the use of dependencies that combine the physical, mechanical and tribological properties of the TS, the criterion of development of processes of the self-organization of subsystems of the second level in TS of diesel engine can be obtained.

For the numerical solution of the differential equation of the energy-mass transfer, a personal computer and the corresponding software were used.

### Data for research

TS of diesel engines—it is the set of physical objects that interact with each other by contact-mechanical means, and at the contact points occurs the breakage of linear velocities of the interacting objects, where the mutual energy-mass transfer takes place^20^.

The simple TS of diesel engines consist of two facilities that operate without lubricating surface with dry abrasive mass. First of all, this concerns the operation of diesel vehicles during the start-up period, and especially at low temperatures. Secondly, even in the presence of lubrication, a regime without lubrication of the surface with dry abrasive mass is possible.

The complex TS of diesel engines are complemented by the active environment, lubricant-coolants and artificially introduced energy sources. In turn, any system is divided into subsystems of the highest order.

The operation of subsystems of diesel engines of any level can be described by the equation of the energy balance and the kinetics of the components that are included in present subsystem and define the nature of the interaction, the internal structural and energy state. In consequence of the interaction between the components of the subsystems in TS of diesel engines, their energy is spent on changing the character of behavior, and another—on changing their internal state^21^. The latter is the energy that is expended to change the kinetics of the interaction between the components of the subsystems in TS of diesel engines and it is the major component of energy balance of the subsystems of the next higher level. The subsystems of each level are characterized by their most informative energy parameters in absolute, specific, gradient and other ratios. Hereafter, they are used to describe the energy, balance and kinetic relations that show the first and the second law of thermodynamics^3^. The combination of the energy relations of functioning of subsystems of different levels is possible, but impractical in the presence of variables of different orders in one equation. Therefore, each subsystem independently from its level should be described by its thermodynamic relation. The lower-level subsystems are described by the equation of energy balance in absolute values or their streams. It is appropriate to describe the high-level subsystems by energy flux density, and the subsystem of higher orders—by specific energy parameters or their gradient expressions.

**Figure 1:**
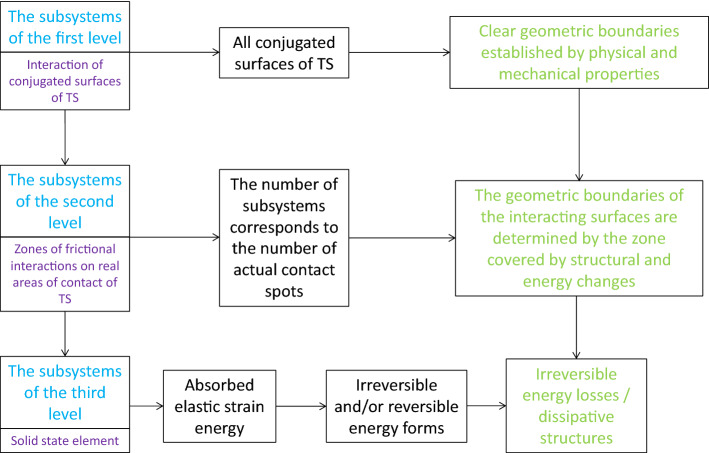
The hierarchy of subsystems of three levels in TS of diesel engines.

The hierarchy of subsystems of three levels in TS of diesel engines is presented graphically in Fig. 1.

In considering the processes of friction and wear that occur in diesel engines, the subsystems of the first level may include the interaction of connected parts with identified geometric borders, different physical and mechanical properties, with the known laws of the external load and movement of each point, as well as the specified interval of velocities in the TS of diesel engines.

The subsystems of the second level in the TS of diesel engines may include areas of friction interactions on the real areas of contact. The number of subsystems of the second level corresponds to the number of spots of actual contact. Geometric boundaries of the interacting objects of present subsystems are defined by the zone, in which the structural and energetic changes are observed.

The subsystem of the third level in the TS of diesel engines may include micro-volume of contact and subcontact zones, covered by the structural and energy changes of triboprocess. In contrast to the subsystems of the first and the second levels in the TS of diesel engines, which provide for interaction, at least of two solid-phase objects, the components of subsystems of the third order is the one solid-phase formation, the change of the structure of which is due to its penetrating flow of energy and matter^1^.

The development of the self-organizing systems is determined by the Glansdorff-Prigogine criterion^3^, under which in any unbalanced system the rate of change of entropy production decreases. Stationary or stable thermodynamic processes are characterized by minimum entropy production.

The energy balance equation for the subsystems of the first level is presented in absolute energy values or in their flows^3^:

1 $$ \overline{F} \cdot \overline{{V_{\kappa } }} = \frac{d}{dt}(Q + W_{m} ) = \dot{Q} + \dot{E}_{m} , $$

where $\overline{F}$ is the average force of contact interaction in the TS; $\overline{{V_{\kappa } }}$ is the average sliding velocity; $Q$ is the amount of heat that stood out in the process of contact interaction between elements of the TS; $W_{m}$ is the mechanical component of the energy balance of the TS; $\dot{Q}$ is the heat flow; $\dot{E}_{m}$ is the flow of mechanical energy in the TS of diesel engines.

The mechanical component of the energy balance of the TS of diesel engines describes the energy cost for surface structural changes and their wear. Its part does not exceed (1.5–3.0) % of the work of friction, thus:

2 $$ \overline{F} \cdot \overline{{V_{\kappa } }} \approx \dot{Q}. $$

This equation shows the balance between work of friction and released thermal energy in the TS of diesel engines or the process of dissipation of the input mechanical energy.

The increase of the produced entropy $\Delta \,S$ determines the mode of operation of the TS of diesel engines. The break-in period is characterized by the lowering of $\Delta \,S$, the steady-state—by its stabilization, and catastrophic wear—by its dramatic growth.

Thermodynamics of the subsystems of the second level in the TS of diesel engines considers the balance relationships, which reflect the transformation of the nondissipative mechanical energy, which can be written as^3^:

3 $$ \frac{{\partial^{2} E_{m} }}{\partial m\partial t} = \frac{{\partial^{2} U}}{\partial m\partial t} + \frac{{\partial^{2} A_{wp} }}{\partial m\partial t} + \frac{{\partial^{2} Q}}{\partial m\partial t} $$

where $U$ is the internal energy; $A_{wp}$ is the work of the wear process; $m$ is the mass of the objects of the contact interaction.

The process of absorption of the elastic internal energy by the contact layers of the TS of diesel engines takes place at the initial moment of frictional interaction or at the break-in period^23^. Upon completion of this process, its intensity drops to zero, periodically renewing as the new contact layers open due to wear.

Consequently, in the steady process of friction, the energy costs for the formation of secondary structures become the values of lower order than the remaining terms of Eq. (3). Then the energy balance equation of the subsystem of the second level in the steady process of wear will look like:

4 $$ \frac{{\partial^{2} E_{m} }}{\partial m\partial t} = \frac{{\partial^{2} U}}{\partial m\partial t} + \frac{{\partial^{2} Q}}{\partial m\partial t}. $$

If the entropy is taken into account in the expression (3), or rather the second variation of the entropy (the excess entropy production)^24^, we would obtain the expression, which is the criterion of possibility of development of processes of the self-organization of subsystems of the second level:

5 $$ \frac{{\partial^{2}_{m} }}{\partial m\partial t} = 2\frac{{\partial^{2} A_{wp} }}{\partial m\partial t} - \left( {\frac{\partial I}{{\partial V}}V + I} \right)^{2} \left( {\delta V} \right)^{2} \frac{{n^{2} }}{{D\,T_{c} }} + \frac{{^{2} }}{{T^{2} B\lambda }}\left( {\frac{\partial \mu }{{\partial v}}v + f} \right)^{2} \left( {\delta V} \right)^{2} - Tgrad\frac{\rho }{T}\left( {\left( {\frac{\partial }{\partial t}\left( {\delta \rho_{g} + \delta v_{gs} } \right)} \right)^{2} + \rho_{g} v_{gs} } \right), \quad \quad \frac{{\partial E_{m} }}{\partial m} \le 0,\;\;\;\frac{{\partial E_{m} }}{\partial t} \le 0, $$

where $T_{c}$ is the cooling temperature of the surface of the part; $T$ is the absolute temperature; $\mu$ is the chemical potentials; $\rho$ is the potential of the dislocation density; $\lambda$ is the thermal conductivity; $f$ is the coefficient of friction; *P* is the pressing force at the contact; $n = \partial N/\partial V$ is the volume concentration of particles; $\partial V$ is the infinitesimal volume; $I$ is the intensity wear; $v$ is the sliding velocity; $D$ is the quasidiffusion coefficient; $B$ is the height of microasperities; $\rho_{g}$ is the average volume density of mobile dislocations; $v_{gs}$ is the average sliding velocity of mobile dislocations.

The criterion for the possibility of development of processes of the self-organization of subsystems of the second level (5) shows that in a real irreversible process there is a decrease in the flow of mechanical energy.

The pressing force at the contact, for example, for the TS of diesel engine “crankshaft-insert” can be defined:

6 $$ P = \left( {P_{g} - \left( {m_{p} + m_{1} + m_{2} \cdot \frac{{l_{c} }}{l}} \right) \cdot j - F_{wp} } \right), $$

where $P_{g}$ is the force of gas pressure on the piston acting along the axis of the cylinder; $m_{p}$, $m_{1}$, $m_{2}$ are the mass of the piston, the connecting rod, which moves back and forth along the axis of the cylinder and the mass of the connecting rod, attributed to its center of mass, which performs a complex movement, respectively; $l$, $l_{c}$ are the total length of the connecting rod and the distance from the center of mass to its upper head, respectively; $j$ is the acceleration of reciprocating motion of the piston; $F_{wp}$ is the force of the wear process in TS of diesel engine.

As a result, expression (5) will take the form:

7 $$ \frac{{\partial^{2} E_{m} }}{\partial m\partial t} = 2\frac{{\partial^{2} A_{wp} }}{\partial m\partial t} - \left( {\frac{\partial I}{{\partial V}}V + I} \right)^{2} \left( {\delta V} \right)^{2} \frac{{n^{2} }}{{DT_{c} }} + \left( {P_{g} - \left( {m_{p} + m_{1} + m_{2} \cdot \frac{{l_{c} }}{l}} \right) \cdot j - F_{wp} } \right)^{2} \left( {\frac{\partial \mu }{{\partial v}} v + f} \right)^{2} \frac{{\left( {\delta V} \right)^{2} }}{{T^{2} B\lambda }} - Tgrad\frac{\rho }{T}\left( {\left( {\frac{\partial }{\partial t}\left( {\delta \rho_{g} + \delta v_{gs} } \right)} \right)^{2} + \rho_{g} v_{gs} } \right),\frac{{\partial E_{m} }}{\partial m} \le 0,\;\;\;\frac{{\partial E_{m} }}{\partial t} \le 0, $$

The external manifestation of the self-organization in the subsystems of the second level is the stabilization of the wear rate, which is determined at the very end after complete stabilization of all the power, thermal and micro-geometrical parameters of the TS of diesel engines.

Thermodynamics of the subsystems of the second level shows the process of transformation of non-thermal component of external flow of mechanical energy into irreversible energy losses and the internal structure and energy transformations that occur in the contact layers at friction.

All the summed up mechanical energy is absorbed by the surface layers of friction bodies in the form of elastic strain energy. As a result, interacting structures correspond to an unbalanced condition.

All possible forms of energy can be divided into two types: reversible and irreversible^3^. Irreversible are those types of forms of energy, which in their development have reached the state at which their subsequent transformation into other forms is potentially impossible. They cannot be involved in the processes of transformation of structures and completely leave the subsystem by spreading in the environment. These species include thermal, acoustic and light energy. Irreversible energy forms are dominant in all tribotechnical and deformation processes.

The ongoing energy-mass transfer within the subsystems of the second level is driven by gradients of temperature, stress, chemical potentials and dislocation density. The direction of the energy-mass transfer is opposite to the vector of gradient of chemical potential, so the decrease in its intensity occurs in the course of the development of the process.

We write the boundary conditions for the differential Eq. (7) in the following form:

8 $$ V = V_{0} ,\;\;m = m_{0} ,\;\;\left. {\left( {\frac{\partial I}{{\partial V}}V + I} \right)^{2} \left( {\delta V} \right)^{2} } \right|_{I = 0} = 0,\;\;\;\left. {\left( {\frac{\partial \mu }{{\partial v}}v + f} \right)^{2} } \right|_{\mu = 0} = 0,\;\;\left. {\frac{{\partial A_{wp} }}{\partial t}} \right|_{t = 0} = 0,\;\;\left. {\left( {\frac{\partial }{\partial t}\left( {\delta \rho_{g} + \delta v_{gs} } \right)} \right)^{2} } \right|_{t = 0} = 0. $$

## Results

The implementation of the energy-mass transfer in the subsystems of the second level presents one of the forms of absorbed dissipation of mechanical energy. The decline in mass due to deformation (wear) also applies to the dissipative phenomena, since the internal energy, which is carried away by wear particles and energy costs, which are intended for the implementation of the wear work, permanently leave the subsystem of the second level.

The equation describing the process of dissipation of the internal energy (7) from the physical point of view—it is the transfer equation in which the first and the second terms describe the processes of surface damage (convective mass transfer), and the last two—processes of the energy-mass transfer, implemented by the gradient relations.

Let us consider expression (7) in more detail, the boundary conditions for it (8) and try to explain the processes that can occur in the interacting surfaces of the TS of diesel engines during their operation.

The first case. If in the TS of diesel engines flow the processes of the self-organization in full, then in the last expression of the value $A_{wp}$ and $I > 0$.

In this case, the expression (7) will present the dependence of the form:

9 $$ E_{m} = f(V,\rho_{g} ). $$

**Figure 2:**
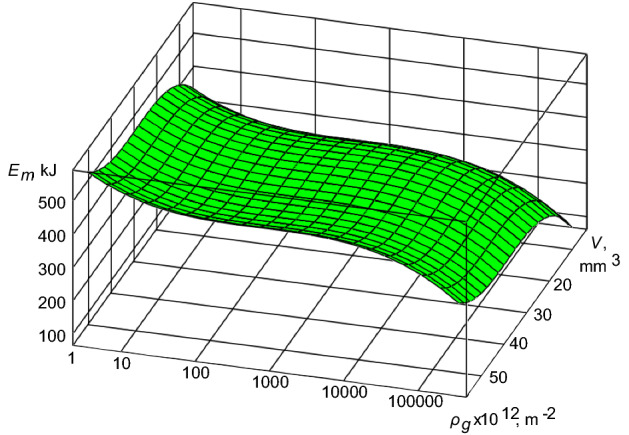
The energy-mass transfer in the TS “crankshaft-insert” for the operating conditions of diesel engine 10D100 during the self-organization processes.

Using the numerical methods for solving differential equations^25^ and taking into account the above, we construct the dependence of the energy-mass transfer (9) for the condition of the processes of self-organization in the TS of the second level on the example of the TS “crankshaft-insert” for the design operating conditions of diesel engine 10D100^21^ (Fig. 2).

As it is seen from Fig. 2, the intense energy-mass transfer occurs with a small dissipation of entropy out of the zone of friction of the TS “crankshaft-insert”. Along with the gradual increase in the density of mobile dislocations, the energy-mass transfer is minimized and directed to the stable unbalanced state. In this case, there is a reduction of entropy from the friction zone into the environment. The volume of the interacting surfaces of materials of the TS, in this case, has little influence on the processes of the energy-mass transfer.

**Figure 3:**
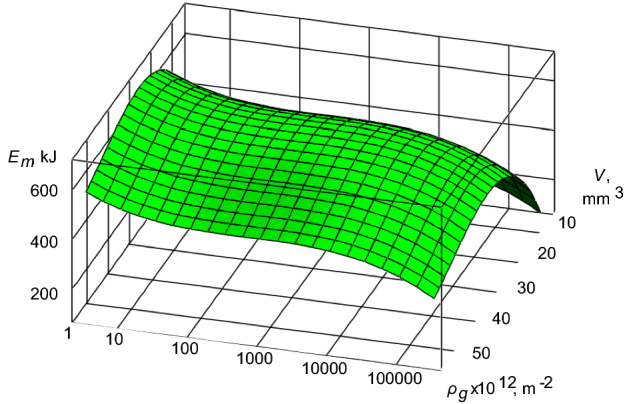
The energy-mass transfer in the TS “crankshaft-insert” for the operating conditions of diesel engine 10D100 during the partial self-organizing processes.

Further, we will consider the second case, which concerns the fact that in the interacting surfaces of the TS of diesel engines of the second level there will proceed partial self-organization processes. In accordance with the Eq. (7), the basic variables will seek to:

10 $$ A_{wp} \to \min ;\,\,\,\,\,\,\,\,I \to \min ; $$

and herewith the Eq. (7) and the boundary conditions (8) will follow the dependence (9). However, through applying the numerical methods for solving differential equations^25^, we will obtain more complicated solution, which can be graphically presented for the TS “crankshaft-insert” during the operation of diesel engine 10D100 and during the partial self-organizing processes as follows (Fig. 3).

Depending on the interacting volume of materials of mating surfaces of the TS “crankshaft-insert” during the partial self-organizing process there are significant deviations of the energy-mass transfer. This indicates a partial decrease in entropy in the friction zone of the TS of diesel engine. The significant increase in the density of mobile dislocations leads to the decrease in the value and the gradual stabilization of the energy-mass transfer that is the evidence of the formation of steady secondary structures on the interacting surfaces of friction.

The third case concerns the assumption that in the TS of diesel engine of the second level there will not occur even partial self-organizing processes, therefore, we conclude that the interacting surfaces of the TS will be damaged in a short time, i.e. the critical conditions in this case will have the following form:

11 $$ A_{wp} \to \max ;\,\,\,\,\,\,\,\,I \to \max ; $$

The latter condition indicates the following: whether the burn-in processes or the processes of excessive wear of the TS of diesel engines. In the first case—we will receive the stabilization of the energy-mass transfer, namely there will occur the transition to normal conditions of the wear process of the TS (operation) with possible following transition to the partial processes of self-organization in them. In the second case—improvement of resource failures, reducing the probability of failure-free operation, reliability and durability of the TS, parts or diesel engines in general.

**Figure 4:**
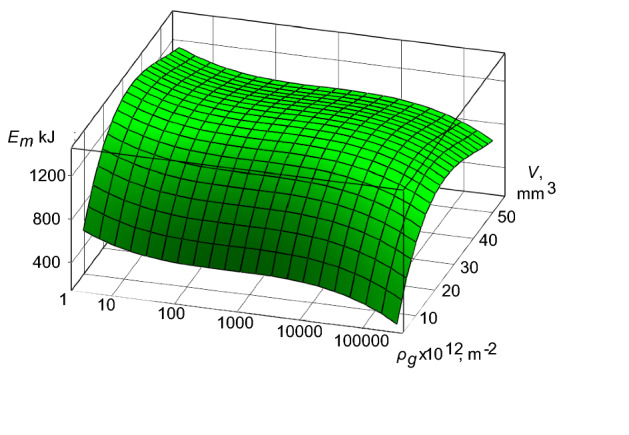
The energy-mass transfer in the TS “crankshaft-insert” for the operating conditions of diesel engine 10D100 during the processes of critical wear of interacting materials.

Taking into account the condition (11) and using numerical methods for solving the expression (7) and the boundary conditions (8), we have obtained the following graphical interpretation (Fig. 4) for the TS “crankshaft-insert” of diesel engines 10D100.

The above graphic dependence indicates the flow of the excessive process of the energy-mass transfer that confirms the condition (11) and the critical values of wear of interacting materials of the TS.

These solutions for the three cases, based on the example of the TS “crankshaft-insert” for the operating conditions of diesel engine 10D100, prove that the expression (7) with the boundary conditions (8) can be considered as the criterion of the possibility of development of processes of the self-organization of subsystems of the second level.

## Discussion

In the work is to establish the criterion of development of processes of the self-organization of subsystems of the second level in TS of diesel engine [expression (7) and the boundary conditions for it (8)]. Based on the proposed criterion and the application of a numerical solution of the differential equation for the TS “crankshaft-insert” of a diesel engine 10D100, the level of the energy-mass transfer was determined in the absence and flow of self-organization processes. For the initial operating conditions of the TS “crankshaft-insert” of a diesel engine 10D100 is characterized by the presence of intense of the energy-mass transfer (Fig. 2). This process can be explained by the running in of interacting surfaces. With an increase in the density of mobile dislocations, the energy-mass transfer tends to a minimum. At the same time, entropy decreases, leading to the appearance of the self-organization processes in the “crankshaft-liner” of diesel engine. On Fig. 3, one can see the flow of partial processes of self-organization, which is confirmed by significant differences in the energy-mass transfer. With an increase in the density of mobile dislocations, the energy-mass transfer is stabilized. The authors suggest this stabilization of the energy-mass transfer occurs as a result of the formation of secondary structures on the interacting surfaces of the “crankshaft-insert” of diesel engine. This is evidence of the flow of partial processes of self-organization in the TS “crankshaft-insert”. The excess level of the energy-mass transfer on the interacting surfaces of the “crankshaft-liner” of diesel engine (Fig. 4) shows a rapid transition from self-organization processes to processes of intense wear and destruction. The processes of intensive wear and destruction for diesel engines are typical for the case of overload, with more than 50% of the nominal parameters and a duration of more than 20 s. The given solutions of the energy-mass transfer in the TS “crankshaft-insert” of a diesel engine 10D100 theoretically confirm the possibility of using expression (7) with boundary conditions as a criterion for the development of processes of self-organization of subsystems of the second level.

A feature of the proposed criterion (7) is the further development of the Glansdorff-Prigozhin criterion, based on a combination of the physical–mechanical and tribological properties of the subsystems of the second level. The proposed criterion differs from the existing ones in that in the Glansdorf-Prigozhin criterion, elements of solid state physics, wear theory and the fundamentals of diesel engine mechanics were applied.

The limitations of this theoretical study are the calculated data, which are given in the reference literature.

The shortcomings of the study include the possibility of calculating only plain bearings in subsystems of the second level of diesel engines. More research is needed to overcome the shortcomings.

The development of this study is to conduct experimental studies of the energy-mass transfer in the TS "crankshaft-liner" of a diesel 10D100. The authors propose to use laser processing of interacting TS materials to change the density of mobile dislocations and the interacting volume. The difficulty that may arise in experimental studies is to register the values of the energy-mass transfer, since the TS "crankshaft-liner" is an open system, which also does not allow taking into account the change in entropy.

## Conclusions

Based on the theories of thermodynamics and change of systems of Glansdorff–Prigogine^3^, it is theoretically determined that in order to reduce the wear rate of the TS it is necessary to provide the flow of the energy-mass transfer process on their contacting surfaces of friction by the gradients of chemical potentials and dislocation density of the interacting materials.

1. Based on the thermodynamic aspect in view of the second variation of entropy (the excess entropy production), we have obtained the expression (7) with the boundary conditions (8) which is the criterion for possibility of development of processes of the self-organization of subsystems of the second level in TS of diesel engine.
2. The external manifestation of the self-organization in such TS of diesel engine is the stabilization of the intensity of wear, which is determined at the very end after after the establishment of equilibrium microgeometric parameters. The latter, perhaps, if the structure of material of the mating surfaces of the TS of diesel engine is in the unbalanced condition. For this, it is necessary to fulfill the condition for reducing the flow of mechanical energy by mass and by the time of contact of the vehicle surfaces in accordance with criterion (7).
3. The resulting expression (7) is a criterion for the possibility of developing self-organization processes. The processes of self-organization of subsystems of the second level of the TS of diesel engines are possible under the condition of a decrease in the flow of mechanical energy both in the contacting mass and in the time of contact of the TS surfaces. As a result, dissipative structures are formed, which may indicate that the subsystem of the second level of the TS of diesel engines would lose stability if there was an increase in the density of mobile dislocations or the wear rate of the TS of a diesel engine.

Further research will consist of conducting experimental studies and comparing them with theoretical ones.

## Data Availability

The datasets used and analysed during the current study available from the corresponding author on reasonable request.
